# Case Report: Delayed Diagnosis of Leprosy-Related Neuropathic Ulcer, Insights from a Case of Delay to Diagnose across Four Clinical Settings

**DOI:** 10.12688/f1000research.157023.2

**Published:** 2025-09-12

**Authors:** Saldy Yusuf

**Affiliations:** 1 Faculty of Nursing, Hasanuddin University, Makassar, Indonesia, 90245, Indonesia

**Keywords:** Neuropathy, Leprosy, Diabetes mellitus, Neuropathic ulcers, Delayed diagnosis Wound care, DMIST tool, Semmes Weinstein Monofilament test.

## Abstract

**Background:**

Neuropathy is common in both Diabetes Mellitus (DM) and Leprosy, often resulting in neuropathic ulcers. Leprosy-related neuropathic ulcers are frequently misdiagnosed as DM-related, causing delays in appropriate care. We report a case which had been misdiagnosed at three different healthcare centers before receiving the correct diagnosis and management.

**Methods:**

The case report adopt the CARE Guidelines and was conducted at the Wound Care Specialist Clinic. Data were collected using a Minimum Data Set (MDS) to capture demographics, health history, and history of treatments. A head-to-toe assessment focused on the eyes, hands, and feet, with neuropathy, confirmed using the Semmes Weinstein Monofilament test, and angiopathy was assessed by palpating the dorsal pedis and posterior tibialis pulse. Wound care interventions consisted of cleansing, debridement, and dressing. Given the similarities between Leprosy-related neuropathic ulcers and DM-related neuropathic ulcers, the DMIST (depth, maceration, inflammation/infection, size, tissue type of the wound bed, type of wound edge, and tunnelling/undermining) tool was used to evaluate wound healing progress.

**Results:**

Anamnesis indicated patient has no DM, with normal blood glucose; however, the patient had neuropathic wounds on her feet, asymmetrical eyebrow distribution, and rashes on her hands and calves, with neuropathy confirmed by a monofilament test—initial treatment involved Cadexomer Iodine powder to control bacterial growth and Honey-based gel to promote granulation. Over 62 days, 11 treatments were administered, with an average dressing change every 5.6 days, which improved the DMIST score from 12 to 4 by the end of observation day.

**Conclusions:**

This case report highlights the significance of distinguishing leprosy-related neuropathic ulcers from those associated with DM to ensure accurate diagnosis and timely treatment. By employing comprehensive assessment tools and targeted wound care interventions, significant improvements in wound healing were achieved, emphasizing the need for greater awareness and clinical vigilance in managing Leprosy-related neuropathic ulcers.

## Introduction

Neuropathy is known as loss of sensation, commonly in both upper and lower extremities. Some term regarding neuropathy, including peripheral neuropathy or peripheral polyneuropathy, encompasses a wide array of disorders that result in damage and dysfunction of the peripheral nervous system’s nerves in various patterns ( Plaut & Weiss, 2021). Epidemiological studies report various ranges of prevalence neuropathy, from a lower rate of 1% to 7% ( Castelli et al., 2020), a moderate rate of 10-15% ( Zhu et al., 2024), and a high rate in Ethiopia 53.6% ( Abdissa et al., 2020) and China 67.6% ( Wang et al., 2023). Different types of diagnostic tests also determine the prevalence. Self-administered questionnaires confirmed 44.4% and 51.1% based on foot examinations ( Perveen et al., 2024). Various diagnostic tests result in clinical challenges in investigating neuropathy.

Diagnosing neuropathy remains challenging. Currently, a triangulation approach that includes clinical evaluations, laboratory tests, and nerve conduction studies (electrodiagnostic test) is the primary method for diagnosing polyneuropathy ( Goedee et al., 2013). Other methods include Diabetic Peripheral Neuropathy, Diagnostic tests including stimulus tests, Screening Scores, Quantitative tests (monofilament, IpTT, Neruothesiometer, Vibratip), and nerve conduction studies ( Galiero et al., 2023). Meanwhile, the diagnostic test for neuropathic Leprosy is challenging. Routine examinations to assess nerve function impairment in Leprosy typically focus on the hands, feet, and eyes. Techniques for evaluating nerve function in leprosy neuropathy include nerve conduction studies (NCS), quantitative sensory tests (QST), assessment of tactile sensitivity in skin lesions using Semmes-Weinstein monofilament testing (MFT) or ballpoint tests, and motor function evaluation through voluntary muscle testing ( Ebenezer & Scollard, 2021). Neuropathy can be diagnosed with advanced tests using electromyography, high-resolution sonography serology, and PCR ( Marques, 2024). The problem in clinical settings is the relative unavailability of standard diagnostic tests, making diagnosing neuropathy challenging. Additionally, patients with neuropathy may not experience discomfort or pain, further complicating diagnosis. Meanwhile, healthcare professionals often seem unaware of neuropathic ulcers, particularly those related to Leprosy.

Another challenge is that gout can indeed lead to ulcerative conditions ( Filanovsky et al., 2015), which may be misinterpreted as neuropathic ulcers. Therefore, integrating anamnesis, thorough clinical assessment, and timely diagnosis is essential to prevent misdiagnosis ( Doghramji et al., 2012). Neuropathy is notably prevalent among patients with DM and Leprosy, frequently leading to the development of neuropathic ulcers. As a result, Leprosy-related neuropathic ulcers are sometimes misdiagnosed as DM-related or as gout related ulcer. This lack of recognition among healthcare professionals leads to frequent misdiagnosis or delayed diagnosis of Leprosy-related neuropathic ulcers. This case report highlights the challenges in accurately diagnosing Leprosy-related neuropathic ulcers, emphasizing the need for timely recognition and a deeper understanding of Leprosy-related neuropathy’s conceptual, clinical, and therapeutic aspects. This is crucial to prevent delays in wound care and improve patient outcomes.

## Case report

The study is a case report which reported based on the CARE Guidelines ( https://www.care-statement.org/checklist). The case was conducted at the Wound Care Specialist Clinic, Griya Afiat, Makassar, East Indonesia. A wound care specialist administered wound care, while a wound care specialist with a doctoral qualification in wound care carried out the healing evaluation. Ethical clearance was obtained from the Ethics Committee of the Faculty of Nursing, Hasanuddin University (75 I/UN4.18.3/TP.01.02/2025).

### Assessment

Data collection was conducted using a Minimum Data Set (MDS) to gather demographic information, health history, and health status, including details of previous treatments. The assessment followed a head-to-toe approach, focusing on the eyes, hands, and feet ( Ebenezer & Scollard, 2021). We inspected the eyebrows for the eyes, comparing the symmetry between the right and left eyes. The inspection was then extended to the hands and feet, comparing the right and left sides. A foot assessment was performed to identify the status of neuropathy and angiopathy. Neuropathy was confirmed through the Semmes Weinstein Monofilament test (10g), applied to the plantar hallux, first metatarsal, third metatarsal, and fifth metatarsal. Angiopathy status was assessed by palpating the dorsal pedis and posterior tibialis pulses.

### Wound care

The wound care interventions included cleansing, debridement, and dressing. We used mineral water and liquid soap for the peri-wound skin and the wound bed for wound cleansing. Sharp debridement and mechanical debridement were performed to remove the callus surrounding the wound. Dressing selection was based on the specific needs of the wound bed. It included three layers: a primary dressing (in direct contact with the wound bed), a secondary dressing (to absorb exudate), and a tertiary dressing (for fixation).

### Wound evaluation

Photographs were taken using a mobile phone without photographic applications to avoid image manipulation. The photos were cropped using the cropping feature in MS Word to remove unnecessary elements while ensuring the observation area (the foot) remained intact. Since the Leprosy-related neuropathic ulcers’ characteristics are similar to those of DM related neuropathic ulcer, we use the DMIST tool for wound healing evaluation. DMIST is a valid healing score for diabetic foot ulcers ( Oe et al., 2020) and is useful for predicting healing ( Tsuchiya et al., 2023). The DMIST tool was utilized to evaluate each wound treatment following cleansing and debridement, which were performed by a certified wound care nurse with a doctoral degree.

### Demographics, medical history, and wound history

The patient is a 48-year-old woman, a high school graduate, and a housewife. She reported no history of Hypertension, Diabetes Mellitus, or Tuberculosis. The patient also stated that no family members have had similar wounds. The onset of the wound occurred in November 2023, beginning with the development of thick, numb calluses. The patient attempted to pinch and peel the calluses using her nails, which caused the wound to enlarge, become red (inflamed), and eventually exude fluid. Following advice from her family, the patient sought treatment at a general clinic in November 2023.

### Treatment history

**Figure 1.**
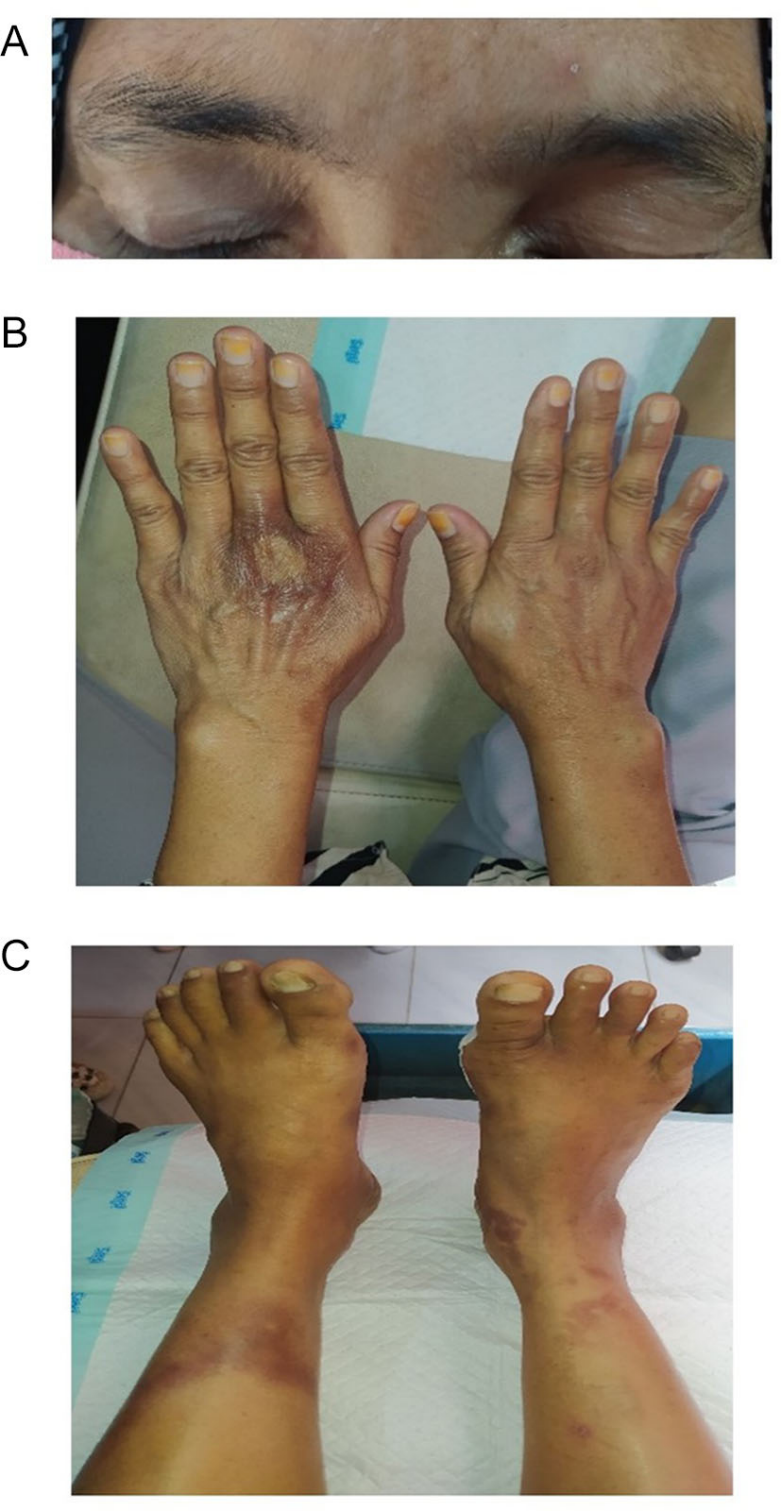
Head to toe assessment. A: We observed an asymmetrical pattern in the patient's eyebrows. B: The inspection revealed a rash on the dorsal side of the left hand. C: Rashes were also found on the left and right tibial areas.

1. **Prior identification of Leprosy status**
  a. **November 2023, Visit to a General Clinic.**
The patient presented to the general clinic with a complaint of a wound on the sole of her foot. During the visit, she reported that she was only interviewed without undergoing any physical examination, including screening for Leprosy. The patient was referred to a hospital for further evaluation.
  b. **November 2023, Visit to the Hospital.**
The patient underwent an examination at the hospital, but no diagnosis of Leprosy was made at that time. The doctor advised the patient not to peel the calluses and recommended using a moisturizer. Wound care included cleansing with NaCl 0.9%, soap, and povidone-iodine and covering the wound with gauze. The patient was instructed to attend three wound care sessions at the hospital before continuing wound care at home.
  c. **February 2024, Visit to a General Practitioner’s Office.**
The patient visited a general practitioner’s office due to the persistence of the wound, along with the development of bruises on her hands and calves. The doctor advised her to avoid prolonged contact with water, detergents, and dishwashing soap. Unfortunately, the doctor suspected the condition was related to gout or rheumatism. The patient was prescribed three types of medications, although she could not recall their names, stating only that one was for muscle cramps. There was no noticeable improvement following this treatment.
  d. **March 2024, Second Visit to the General Clinic.**
Due to the lack of improvement after the consultation at the general practitioner’s office, the patient returned to the same general clinic she visited in November 2023 for a second evaluation. The doctor observed the wound and measured her blood sugar, 85 mg/dL. The doctor explained that the wound required regular care and referred the patient to a specialized wound care clinic. The prescribed treatments included paracetamol, an analgesic, and gentamicin ointment.
2. **After identification, Leprosy status**
  a. **March 2024, Visit to a Wound Care Specialist Clinic.**
The patient visited the Wound Care Specialist Clinic, where anamnesis, a physical examination, and a monofilament test were conducted. The patient’s weight was 43 kg, blood pressure 90 mmHg, blood glucose 71 mg/dL, cholesterol 196 mg/dL, and uric acid 5.1 mg/dL ( Table 1). Anamnesis confirmed that the patient had no history of diabetes mellitus, and her random blood glucose was within the normal range. However, she had neuropathic wounds on her feet. A head-to-toe examination revealed asymmetrical eyebrow distribution, rashes on the dorsal side of her hands, and rashes on both calves ( Figure 1). The monofilament test confirmed neuropathy in the right foot. Palpation of the dorsalis pedis and posterior tibialis pulses was positive in both the left and right feet ( Table 2). Based on these findings, the patient was referred to a health clinic for further referral to a specialized facility to diagnose Leprosy.
  b. **March 2024, Third Visit to the General Clinic.**
The patient returned to the general clinic (third visit), bringing the monofilament test results from the Wound Care Specialist Clinic. The doctor confirmed the findings using a rolled tissue test on the rash-covered areas but obtained negative results. The patient was then referred to a dermatological center for further diagnostic evaluation.
  c. **March 2024, Visit to the Dermatological Center.**
The attending physician conducted a series of tests at the dermatological center. The first test used tissue, followed by a skin smear from the area behind the patient’s ears and both calves. The patient did not receive medication or wound care and was referred to the health clinic for further evaluation.
  d. **March 2024, Fourth Visit to the General Clinic.**
The patient returned to the health clinic (fourth visit) with results from the dermatological center, confirming Leprosy diagnosis. No medication or wound care was provided at the clinic. The patient was referred to a public health center for treatment.
  e. **March 2024, Visit to the Public Health Center.**
The patient visited the public health center, where the doctor performed a ballpoint test and confirmed neuropathy. The doctor explained that the patient would undergo one year of treatment for Leprosy and was advised to continue wound care independently at home.
  f. **March 2024, Return to the Wound Care Clinic.**
The patient returned to the wound care clinic to receive wound care until complete healing was achieved.

**Table 1.** Demographic characteristics and health status.

Demography characteristics	Finding
Age	48 years
Sex	Female
Education background	Literate
Occupation	Housewife
Health history	• No Diabetes Mellitus • No Hypertension • No Tuberculosis
Laboratory finding	• Blood sugar: 71 mg/dl • Cholesterol: 196 mg/dl • Uric Acid: 5.1 mg/dl

**Table 2.** Foot assessment between right and left foot.

Foot assessment	Right foot	Left foot
**Angiopathy test (Palpation)**		
Dorsalis Pedis	Present	Present
Posterior Tibilias	Present	Present
**Neuorpathy test (Monofilament)**		
Plantar hallux	Present	Present
1 ^st^ metatarsal	Absent	Present
3 ^rd^ metatarsal	Present	Present
5 ^th^ metatarsal	Absent	Present

### Wound care process

Wound care was administered by a wound care specialist and involved three modalities of intervention: cleansing, debridement, and dressing. Mineral water and liquid soap were used for wound cleansing. Sharp debridement was performed to remove necrotic tissue and calluses surrounding the wound, in combination with mechanical debridement.

During the first week, Cadexomer Iodine powder was applied as the primary dressing to control bacterial growth at the wound bed, with foam as the tertiary dressing. From the second week onward, honey-based gel was applied to stimulate granulation tissue growth, low-adherent dressing was used as the secondary layer, and gauze was applied during the final phase of care. Adhesive tape was used for fixation ( Table 3).

**Table 3.** Wound care process for each visitation day.

Visitation	Date	Wound dressing		
		Primary dressing	Secondary dressing	Tertiary dressing
1	March, 20 ^th^ 2024	Cadexomer Iodine	Foam	Adhesive tape
2	March, 22 ^nd^ 2024	Cadexomer Iodine	Foam	Adhesive tape
3	March, 26 ^th^ 2024	Cadexomer Iodine	Foam	Adhesive tape
4	March, 29 ^th^ 2024	Honey based gel	Low Adherent	Adhesive tape
5	April, 4 ^th^ 2024	Honey based gel	Low Adherent	Adhesive tape
6	April, 16 ^th^ 2024	Honey based gel	Low Adherent	Adhesive tape
7	April, 23 ^rd^ 2024	Honey based gel	Low Adherent	Adhesive tape
8	April, 30 ^th^ 2024	Honey based gel	Low Adherent	Adhesive tape
9	May, 7 ^th^ 2024	Honey based gel	Low Adherent	Adhesive tape
10	May, 14 ^th^ 2024	Honey based gel	Low Adherent	Adhesive tape
11	May, 21 ^st^ 2024	Honey based gel	Gauze	Adhesive tape

**Figure 2.**
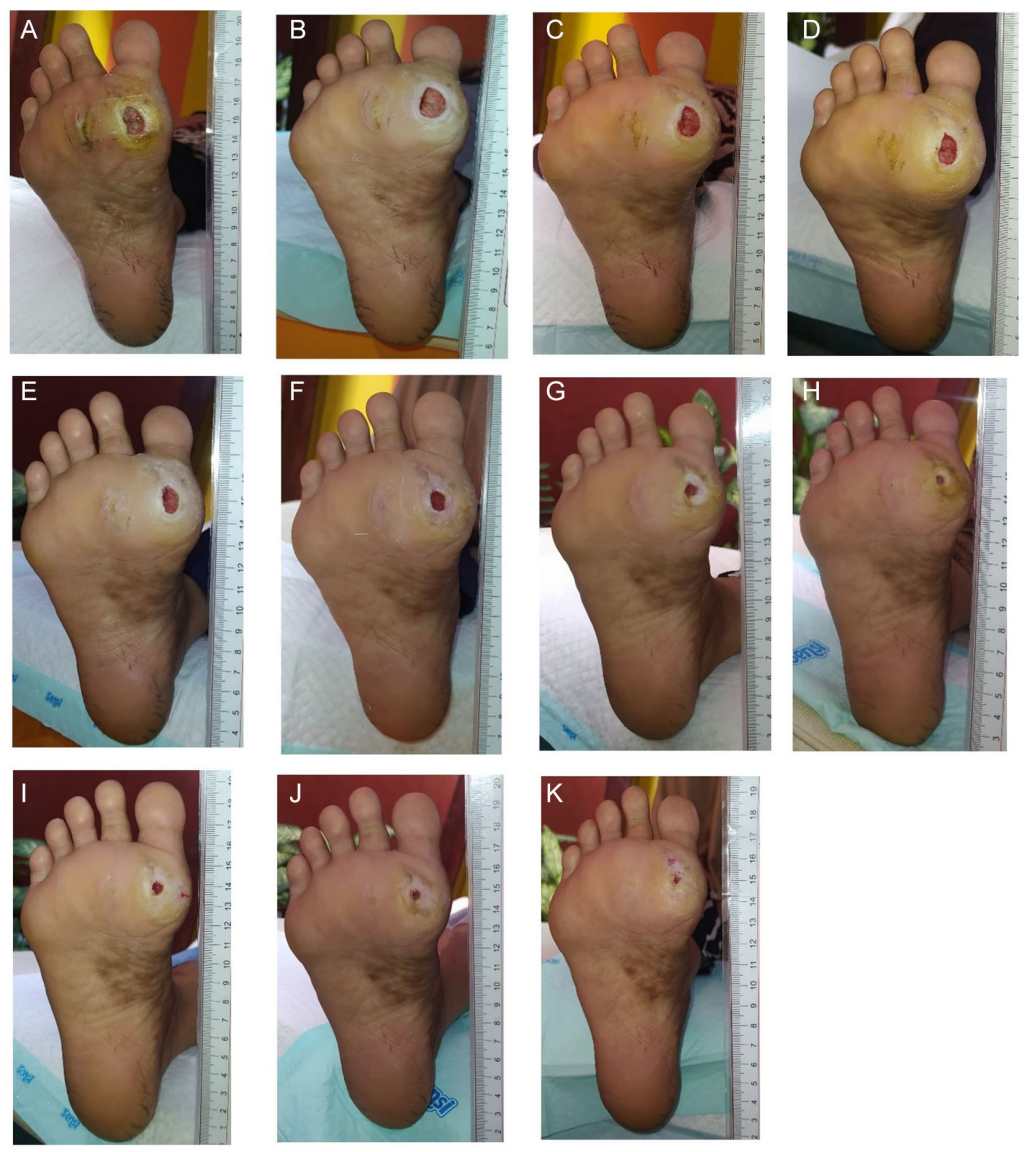
Documentation of wound healing process. A. March, 20 ^th^ 2024. B. March, 22 ^nd^ 2024. C. March, 26 ^th^ 2024. D. March, 29 ^th^ 2024. E. April, 4 ^th^ 2024. F. April, 16 ^th^ 2024. G. April, 23 ^rd^ 2024. H. April, 30 ^th^ 2024. I. May, 7 ^th^ 2024. J. May, 14 ^th^ 2024. K. May, 21 ^st^ 2024.

Wound care began on March 20 and continued until May 21, 2024, spanning 62 days with a total of 11 treatments. The average dressing change interval was 5.6 days. The patient’s total DMIST score was 12 at the first treatment, decreased to 9 in the second month, and reached four at the end of the treatment period ( Figure 2).

### Clinical challenges

The patient works as a household assistant, remaining physically active, which often causes the wound dressing to be wet. As a result, the patient was educated on self-care for the wound, including how to replace the dressing when it became wet. The patient was instructed to clean the wound, apply ointment, and cover it with gauze independently when needed.

### Patient perspective

The patient reported visiting three healthcare facilities, none establishing a diagnosis of Leprosy. The first facility considered it a regular wound, while the third suspected gout—none of the three healthcare facilities performed any tests on the rashes on her hands or feet. The patient stated that the diagnosis of Leprosy was confirmed only after visiting the wound care clinic, where a monofilament test was conducted, followed by a referral back to the healthcare clinic. Only after receiving the monofilament test results was a tissue test performed at the healthcare clinic, and the patient was subsequently referred to the dermatological center for further evaluation. The patient responded to the diagnostic test results with acceptance but mentioned that she would not disclose her leprosy diagnosis to her husband.

### New finding

An exciting aspect of this case is that, despite having a neuropathic ulcer, the patient remained active, walking and working without developing edema or erythema in the affected foot during the two-month treatment period. This is in stark contrast to neuropathic ulcers in patients with DM, where the absence of offloading typically leads to edema, erythema, and local inflammation. Additionally, the healing rate was relatively rapid, with complete recovery achieved in just 62 days, which differs significantly from the prolonged healing process often observed in diabetic patients with similar wounds.

## Discussion

### Diagnostic challenge

The interesting aspect of this case lies in the healthcare facility’s oversight in recognizing and diagnosing the existing neuropathic wounds as a sign of leprosy. Proper diagnosis of neuropathy requires a thorough assessment of patient history, physical examination, and laboratory test results. Early-stage neuropathy is characterized by progressive sensory changes, including the loss of sensation, numbness, pain, or burning sensations in a “stocking and glove” distribution of the extremities. This can progress to proximal numbness, distal weakness, or muscle atrophy ( Castelli et al., 2020). The clinical manifestations of diabetic peripheral neuropathy (DPN) range from asymptomatic to painful neuropathic symptoms ( Abdissa et al., 2020). Common clinical features of DPN include bilateral limb pain, numbness, and paresthesia, which may lead to the development of foot ulcers ( Zhu et al., 2024). The pain pattern in DPN is often described as “glove and stocking” with burning, electric, sharp, or dull aching sensations of varying intensities ( Chang & Yang, 2023). In this case, we observed clinical signs of numbness in the hands and soles of the feet. The absence of pain in the neuropathic wounds is consistent with the characteristics of Leprosy-related neuropathic ulcers ( Khadilkar et al., 2021). and the monofilament test was negative. Skin lesions tend to be hypoesthetic or anesthetic and are typically found on the back, buttocks, trunk, face, and earlobes ( Khadilkar et al., 2021).

### Monofilament TEST as key

In this case, our suspicion arose from the presence of neuropathic wounds without any history or status of DM. Inspecting the eyes, hands, and feet raised concerns about Leprosy. Our approach aligned with recommendations for assessing nerve function impairment in leprosy neuropathy, which typically focuses on the hands, feet, and eyes ( Ebenezer & Scollard, 2021); we then confirmed these findings through a monofilament test. The monofilament test is recognized as a diagnostic tool for leprosy neuropathy ( Frade et al., 2021), being straightforward, practical, cost-efficient, and demonstrating high accuracy in diagnosing clinical Leprosy ( Frade et al., 2022); in Indonesia, the monofilament test is also used to diagnose leprosy-related neuropathy. 95.2% of patients reported experiencing neuropathy symptoms, with 52.4% having symptoms for one year or less and 52.4% exhibiting neuropathy primarily in the lower bilateral extremities ( Dalimunthe et al., 2023). Unfortunately, monofilament tests are not yet widely available in Indonesia, which may contribute to delays in diagnosing leprosy-related neuropathy. Nevertheless, the IpTT examination can serve as an alternative for diagnosing neuropathy ( Basir et al., 2020) when the monofilament test is unavailable.

### Healing process

In this case, we performed sharp debridement to remove the callus surrounding the ulcer. For infection control, we applied Cadexomer Iodine. Cadexomer iodine, an advanced iodine formulation utilizing microbead technology, offers a unique approach to wound care by effectively managing biofilm and exudate while demonstrating superior desloughing capabilities ( Ruke & Savai, 2019). At the end of the second week of treatment, we used a honey-based gel to promote granulation tissue formation. Based on our clinical experience, honey accelerates the growth of granulation tissue. A randomized controlled trial (RCT) is currently investigating the role of honey in ulcer healing in Leprosy ( Udo et al., 2024). In this case, the wound healed within 62 days, similar to previous studies. The median healing time for wounds was 75 days for neuropathic ulcers, compared to 136 days for ischemic ulcers and 171 days for neuroischemic ulcers ( Singh et al., 2023).

### Lesson learning

An important learning point from this case is the failure to detect Leprosy. The patient had been experiencing neuropathic wounds for six months and had visited four healthcare facilities, yet none recognized the neuropathic wounds as a consequence of Leprosy. This aligns with classic reports that leprosy patients experience a diagnostic delay of approximately 1.8 years (ranging from 0.2 to 15.2 years) ( Lockwood & Reid, 2001). Another noteworthy issue is that, according to the patient, none of the four healthcare facilities performed a physical examination, relying only on routine interviews. Several factors contributed to the delayed diagnosis, including a lack of awareness among healthcare workers regarding the symptoms of Leprosy, low clinical suspicion from physicians, and delayed referral to specialist facilities ( Khadilkar et al., 2021). The diagnosis of Leprosy was eventually made after the patient was referred to a Private Wound Care Clinic, despite patient have to pay which her own budget, since it uncover by national insurance ( Oe, 2024). Current case also highlights that health literacy not only important for patient with chronic disease, but also healthcare professional, to avoid delay in clinical decision ( Kadar, 2024).

## Conclusion

This case report provides valuable insights into the delayed diagnosis of leprosy-related neuropathic ulcers across four healthcare settings. It highlights the critical need for heightened clinical awareness and timely diagnosis to prevent prolonged suffering and complications. Accurate diagnosis and appropriate wound management play a pivotal role in promoting the healing process of leprosy-related neuropathic ulcers. Addressing the factors contributing to diagnostic delays can improve patient outcomes and reduce the burden of undiagnosed Leprosy in clinical practice.

## Consent to publish

A written informed consent was obtained from the patient for the publication of this case report. Patient also received a thorough explanation regarding the purpose of her data. Additionally, we adhered to the principles outlined in the Declaration of Helsinki ( https://shorturl.at/2KDfV).

## Data Availability

No data associated with this article. Figshare: Case Report: Delayed Diagnosis of Leprosy-Related Neuropathic Ulcer, Insights from a Case of Delay to Diagnose across Four Clinical Settings: A Case Report DOI: https://doi.org/10.6084/m9.figshare.27123441.v1 ( Yusuf, 2024). The project contains the following reporting guidelines:
• CARE Checklist Data are available under the terms of the Creative Commons Attribution 4.0 International license (CC-BY 4.0).
