# Supplementary material for: Case Report: Delayed Diagnosis of Leprosy-Related Neuropathic Ulcer, Insights from a Case of Delay to Diagnose across Four Clinical Settings
Source: F1000Res. 2025 Sep 12;13:1211. Originally published 2024 Oct 11. [Version 2] doi: 10.12688/f1000research.157023.2 (PMC12504931; doi:10.12688/f1000research.157023.2)
Supplement: Peer review history

# Reviewer report: Reviewer response for version 2

**DOI:** 10.5256/f1000research.187888.r413765

**Published:** 2025-10-07

**Authors:** Paul Alumbugu Tsaku

**Affiliations:** 1 The Leprosy Mission Nigeria, Abuja, Nigeria

I have no further comments.

Are enough details provided of any physical examination and diagnostic tests, treatment given and outcomes?

Yes

Is the case presented with sufficient detail to be useful for other practitioners?

Yes

Is sufficient discussion included of the importance of the findings and their relevance to future understanding of disease processes, diagnosis or treatment?

Yes

Is the background of the case’s history and progression described in sufficient detail?

Yes

Reviewer Expertise:

Public Health

I confirm that I have read this submission and believe that I have an appropriate level of expertise to confirm that it is of an acceptable scientific standard.

# Reviewer report: Reviewer response for version 1

**DOI:** 10.5256/f1000research.172417.r379894

**Published:** 2025-05-03

**Authors:** Rayaz Ahmed Malik

**Affiliations:** 1 Weill Cornell Medicine-Qatar, Qatar Foundation, Education City, Qatar

- This is an instructive case report with important learning outcomes on the missed diagnosis of leprosy related foot ulceration despite multiple visits to different health centres.
- Use of the monofilament for evaluating diabetic neuropathy was in fact originally adopted from its use in evaluating sensory loss in patients with leprosy in India.
- It is insufficient to simply state the 'The patient returned to the health clinic (fourth visit) with results from the dermatological center, confirming Leprosy diagnosis'.
- What tests were undertake, what were the outcomes?
- In the discussion:The absence of pain in the neuropathic wounds is consistent with the characteristics of Leprosy-related neuropathic ulcers ( Khadilkar et al., 2021). and the monofilament test was negative. The sentence on monofilament is out of place.
- Did the patient have any neuropathic symptoms?

Are enough details provided of any physical examination and diagnostic tests, treatment given and outcomes?

No

Is the case presented with sufficient detail to be useful for other practitioners?

Yes

Is sufficient discussion included of the importance of the findings and their relevance to future understanding of disease processes, diagnosis or treatment?

Yes

Is the background of the case’s history and progression described in sufficient detail?

Yes

Reviewer Expertise:

Diagnosis of peripheral neuropathy

I confirm that I have read this submission and believe that I have an appropriate level of expertise to confirm that it is of an acceptable scientific standard.

# Reviewer report: Reviewer response for version 1

**DOI:** 10.5256/f1000research.172417.r345552

**Published:** 2024-12-26

**Authors:** Paul Alumbugu Tsaku

**Affiliations:** 1 The Leprosy Mission Nigeria, Abuja, Nigeria

This is a well-written case study. However, I have some comments for the author to address:

 1. The abstract should mention that the case was misdiagnosed at three previous centers before the current diagnosis and management.

 2. The author places significant emphasis on differentiating between diabetic mellitus (DM) ulcers and leprosy-related ulcers. However, other previous misdiagnoses, such as gout, should also be discussed in the literature review.

 3. It is more appropriate to refer to the Minimum Data Set (MDS) rather than the Minimum Data 'Sheet.'

 4. In the wound evaluation section, please further describe how the DMIST tool was used for assessing the wound.

 5. Although the author indicated that consent was obtained from the patient in accordance with the Helsinki Declaration, there is no mention of whether ethical clearance was obtained or a justification for why it was not needed.

 6. In the acknowledgment section, the author uses the word "we" to acknowledge contributors, but since there is only one author, it would be more appropriate to use the singular form. 

 Thank you for considering these suggestions.

Are enough details provided of any physical examination and diagnostic tests, treatment given and outcomes?

Yes

Is the case presented with sufficient detail to be useful for other practitioners?

Yes

Is sufficient discussion included of the importance of the findings and their relevance to future understanding of disease processes, diagnosis or treatment?

Yes

Is the background of the case’s history and progression described in sufficient detail?

Yes

Reviewer Expertise:

Public Health

I confirm that I have read this submission and believe that I have an appropriate level of expertise to confirm that it is of an acceptable scientific standard, however I have significant reservations, as outlined above.

# Response

**Published:** 2025-09-08

**Authors:** Saldy Yusuf

**Affiliations:** Faculty of Nursing, Hasanuddin University, Makassar, Sout Sulawesi, Indonesia

Thank you for the reviewers’ valuable feedback. We have addressed the comments as follows:

 Abstract: We have revised the abstract to emphasize that the uniqueness of this case lies in the fact that the wound status was misdiagnosed at three different healthcare facilities before the correct diagnosis and management were established.

 Background: We have added clarification that, in clinical practice, neuropathic ulcers caused by leprosy can be mistaken for other conditions, such as gout-related ulcers, thereby highlighting the importance of accurate diagnosis.

 Methods: We have included information regarding the ethical clearance obtained for this study and provided a detailed description of how the DMIST tool was applied by a certified wound care nurse to assess the wound.

 Terminology: We have revised the term *Minimum Data Sheet* to *Minimum Data Set* for accuracy.

 Acknowledgment: We have corrected the wording by replacing *we* with *I* to appropriately reflect the single authorship.
